# A comparative assessment and economic implications of physical, chemical, and enzymatic treatments of lignocellulosic waste

**DOI:** 10.1186/s40643-025-00955-9

**Published:** 2025-10-17

**Authors:** Pavithra Pari, Nicky Eshtiaghi, Maazuza Othman, Sankar Ganesh Palani

**Affiliations:** 1 Department of Biological Sciences, Birla Institute of Technology and Science, Pilani, Hyderabad Campus, Jawahar Nagar, Kapra Mandal, Medchal District, Telangana 500078 India; 2 Department of Chemical and Environmental Engineering, School of Engineering, RMIT University, GPO BOX 2476, Melbourne, VIC 3001 Australia

**Keywords:** Lignin reduction, Cost-effectiveness, Anaerobic digestion, Ultrasonication, Enzymatic treatment, Sustainable biorefinery

## Abstract

A significant portion of the organic waste generated globally comprises garden and agricultural waste, which are primarily lignocellulosic and recalcitrant, making them unsuitable substrates for biorefineries. This investigation focuses on the effect of eight different treatments on grass clippings, providing a comprehensive comparison of mechanical combined with physical, chemical, and biological treatment methods. Additionally, it examines the economic implications of stirring during treatment. The effects of these treatments on grass clippings were assessed using Van Soest Fiber Analysis, Scanning Electron Microscopy (SEM), X-Ray Diffractometry (XRD), and Fourier Transform Infrared Spectroscopy (FTIR). The influence of stirring was explored by repeating the top four treatments, which achieved maximum lignin reduction, with and without stirring. Alkaline treatment with 0.9% NaOH at 37 °C achieved a 58% reduction in lignin content while preserving cellulose and hemicellulose. Subsequent effective treatment methods included ultrasound, hydrothermal, and ammonia treatments. Although acid thermal hydrolysis did not reduce lignin, it significantly removed hemicellulose. Stirring’s impact on lignin removal efficiency was found to be minimal with insignificant *p*-values from Welsch’s t-test. Overall, alkaline treatment at 37 °C emerges as a cost-effective and scalable approach for converting lignocellulosic waste into valuable products. This study uniquely contributes to the optimisation of lignocellulosic waste management by providing a comparative analysis of various treatments of lignin-rich grass clippings and evaluating the role of stirring in enhancing treatment efficiency and reducing costs, thereby paving the way towards sustainable waste valorisation.

**Graphical abstract:**

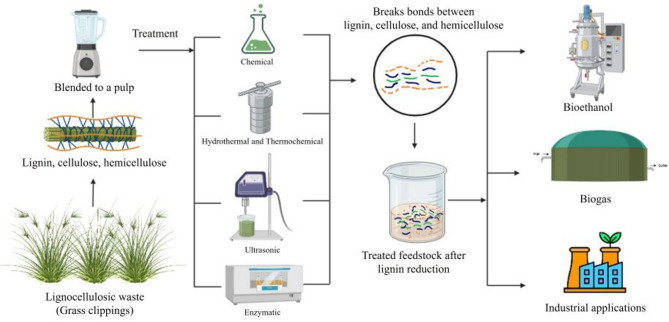

## Introduction

Approximately 140 gigatons of lignocellulosic waste are generated worldwide each year (Devi et al. 2023), including natural, municipal, and industrial sources, most of which contain grass clippings. Burning or composting has been the primary method for the disposal of lignocellulosic waste, but this leads to significant greenhouse gas emissions. Different methods have been employed for the valorisation of grass clippings into biogas, bioethanol, or other valuable products. Biomethanation of grass clippings can be a sustainable and cost-effective alternative, but it is faced with challenges due to the recalcitrance posed by its lignin contents. It has also been suggested that grass clippings can be added to food waste as a co-substrate to act as a buffer and to improve the process stability, which is often affected by low pH caused by the accumulation of volatile fatty acids (Chakraborty et al. 2022).

Various treatment methods are being employed to degrade the lignin content before utilizing lignocellulosic waste as a substrate for valorisation. (Gao et al. 2022) These methods are broadly categorized into physical, chemical, and biological methods. The commonly used physical treatments are either thermal or mechanical. Thermal treatments include different forms of heating, such as the hydrothermal method, liquid hot water treatment, and steam explosion (Liczbiński et al. 2022; Wells et al. 2021; Phuttaro et al. 2019; Xie et al. 2022; Vakalis et al. 2022; Lizasoain et al. 2016; Zhou et al. 2017). The different mechanical treatment methods include using different equipment, from a kitchen blender to a crossflow grinder, ball mill, hammer mill, mounted mower, disc mower, and excoriator (Arce and Kratky 2022; Heller et al. 2023; Tsapekos et al. 2017; Feng et al. 2018). Chemical treatments include acid, alkaline, and organosolv treatments. Acid treatment utilizes sulphuric, hydrochloric, and acetic acids in diluted concentrations (Semeraro et al. 2021; Antonopoulou et al. 2020; Karki et al. 2022). Alkaline treatment utilizes hydroxides of sodium, potassium, and calcium, or aqueous ammonia solution (Saidkhilli and Dhanya 2025; Pinpatthanapong et al. 2022; Narinthorn et al. 2019; Khor et al. 2018; Jiang et al. 2017; Kullavanijaya and Chavalparit 2020; Kang et al. 2018; Chang et al. 2017). Aqueous ethanol in combination with sulphuric, hydrochloric, and acetic acids or sodium hydroxide is utilized for the organosolv treatment (Jomnonkhaow et al. 2022). Biological treatments involve the usage of enzymes either from natural sources, such as black rot fungi, or that are commercially available, such as hemicellulase, lignin peroxidase, laccase, and cellulase (Jin et al. 2015; Saritpongteeraka et al. 2020; Giacobbe et al. 2018; Frydendal-Nielsen et al. 2016). Some other treatment methods also include ultrasonication, laccase-assisted sodium chlorite (LASC) pretreatment (Saini et al. 2023), and ensilage.

While numerous studies have investigated individual pretreatment methods for lignocellulosic biomass, most have focused on isolated techniques such as dilute acid hydrolysis, alkali treatment, steam explosion, or biological methods, often applied to different substrates and under varying conditions (e.g., Arce and Kratky 2022; Chakraborty et al. 2022). A comprehensive comparison of various techniques applied to a single substrate is lacking. The existing published studies were often focused on isolated approaches with limited comparisons. Also, in conventional chemical treatments, stirring is typically employed to enhance the reagent-substrate interactions, thereby improving lignin reduction efficiency. However, the necessity of stirring has not been extensively studied; hence, its cost-effectiveness remains unexplored. Although it is intuitively assumed to be beneficial, stirring incurs significant costs due to the requirements for specialized equipment, increased energy consumption, and maintenance. Conversely, if stirring is unnecessary, treatment could be performed by merely immersing the substrate in the chemical. However, the trade-off between the cost of stirring and lignin reduction has to be established and proven as economically viable.

To address this gap, this study investigates multiple pretreatments in order to optimize treatment efficacy for grass clippings, a prevalent and potentially valuable feedstock for biofuel production and sustainable energy generation. This investigation focused on two primary goals. First, to the knowledge of the authors, this is the first study that conducts a comprehensive comparative assessment of physical, chemical, hydrothermal, thermochemical, and biological pretreatment methods applied to a single, underutilized feedstock: grass clippings. This allows for a direct comparison across techniques under consistent conditions. Second, it uniquely investigates the role and economic feasibility of stirring in chemical pretreatments, an overlooked but critical factor for industrial scaling. By filling these two key gaps, this study contributes original insights that extend beyond existing works, offering a more holistic framework for optimizing lignocellulosic waste valorisation and advancing its potential for biofuel and sustainable energy applications.

## Methodology

### Collection of grass clippings and selection of treatment method

The clippings of grass (*Digitaria sanguinalis*), typically measuring 6–8 mm, were collected from the mower used to trim the lawns of BITS Pilani, Hyderabad Campus, India. The collected grass clippings were ground with a home blender to obtain a homogeneous mixture before being subjected to other treatment methods. The single batch of ground grass clippings was stored at 4 °C and used throughout the complete study to eliminate batch-to-batch variability.

**Fig. 1:**
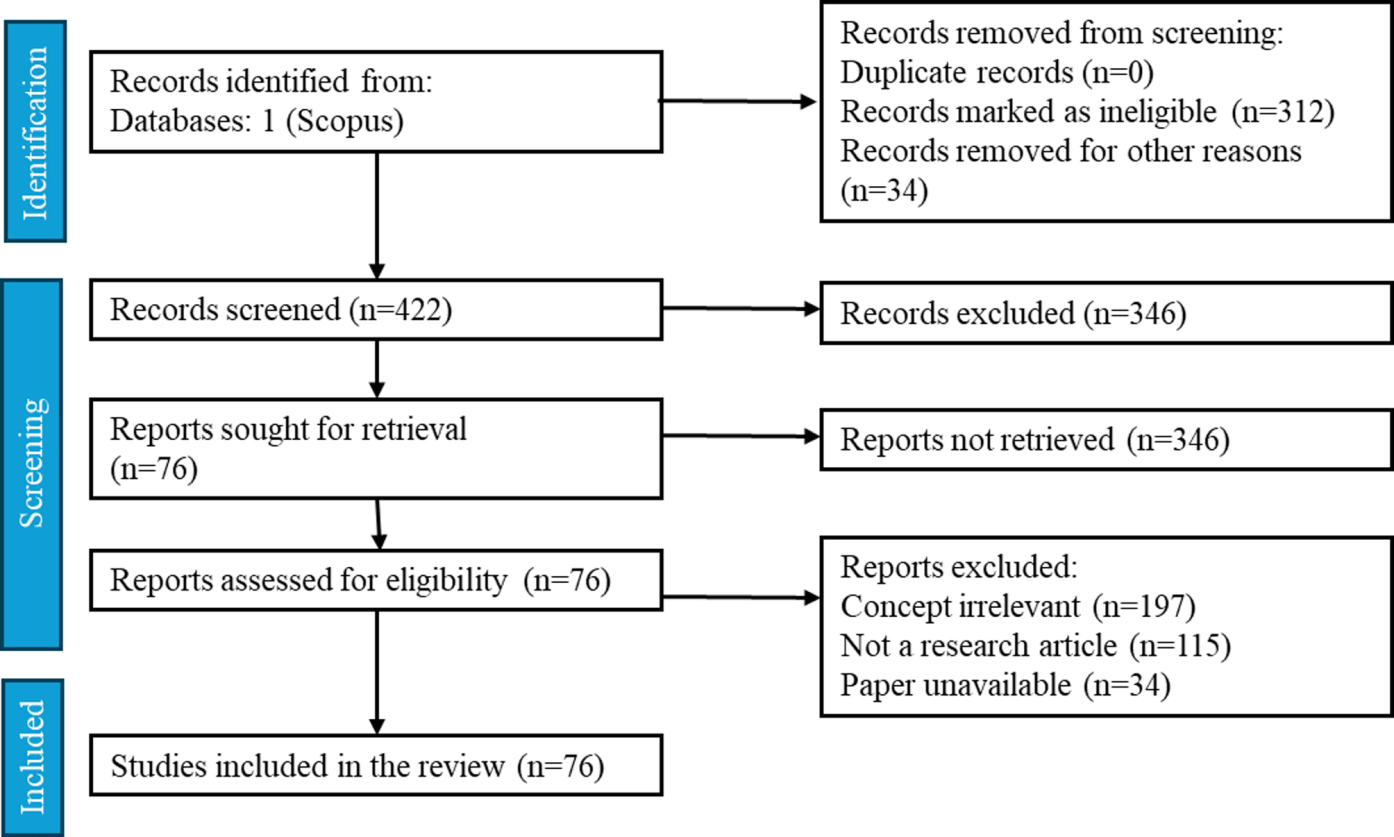
PRISMA framework outlining the identification of studies pretreatment parameters selection

A systematic literature review was conducted to identify the most widely implemented pretreatment methods for lignocellulosic waste, following the PRISMA framework (Fig. 1). Scopus was chosen as the primary database, and the keywords used were: pretreatment, lignocellulosic waste, and anaerobic digestion. A total of 76 papers were identified and screened for relevance. The most reported concentrations, temperatures, and durations were identified from the literature, and the pretreatment parameters for this study were finalized by selecting a range that spanned two increments below and above these commonly used values.

Based on the above literature review, the following eight treatment methods were selected and studied in this investigation: (1) hydrothermal, (2) ultrasonication, (3) enzymatic, (4) sodium hydroxide and (5) sulphuric acid at 37 °C, (6) sodium hydroxide and (7) sulphuric acid at HPHT (High Pressure: 103 kPa, High Temperature: 120 °C), and (8) aqueous ammonia treatment.

### Treatment of grass

**Fig. 2:**
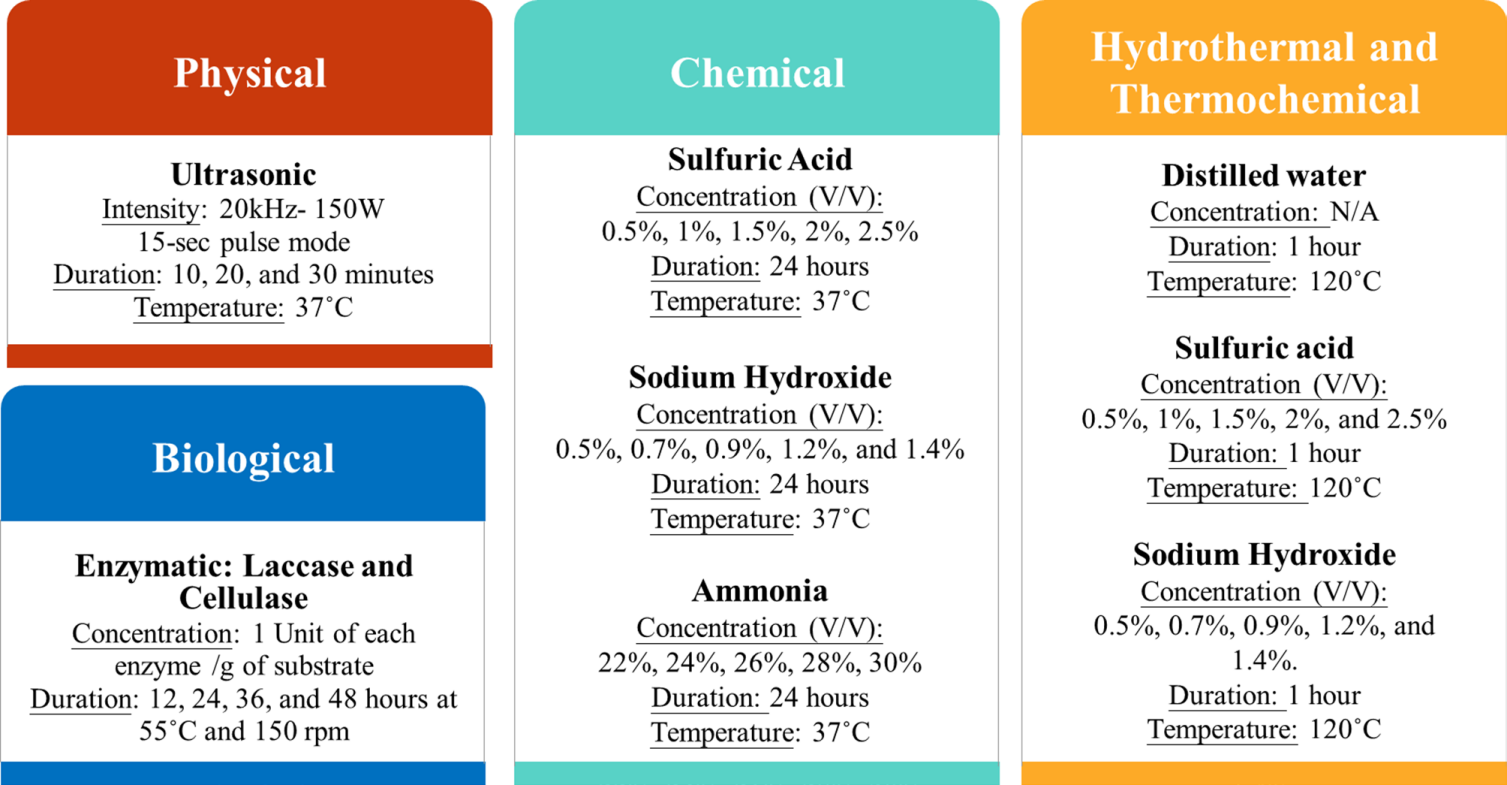
Experimental conditions

The mixing ratio for treatment was 1: 10 (10 g of ground grass in 100 ml of treatment solution). The grass clippings were ground into a pulp with a kitchen blender and used as it is in the treatments that were conducted in triplicate. For hydrothermal treatment, 10 g of grass was mixed in 100 ml of distilled water and heated at 120 °C for 60 min at a pressure of 103 kPa, followed by filtration. Ultrasonication was performed at a power density of 150 Watts, at 20 Hz frequency, with a 15-second pulse and 10-second pause. 10 g of grass was ground into a pulp and mixed in 100 ml of distilled water, and was ultrasonicated at 10, 20, and 30 min. Solutions of sulphuric acid, sodium hydroxide, and aqueous ammonia were used for the chemical treatment at different concentrations and temperatures, as summarized in Fig. 2. The experiments at 37 °C were conducted for 24 h, whereas the hydrothermal and thermochemical experiments were conducted for 1 h. After chemical treatment, the samples were filtered and washed three times to prevent chemical interference during analysis (Wen et al. 2015). All the samples were dried at 60 °C for 6 h before further analysis. An enzyme cocktail (Masran et al. 2020) was prepared by mixing Cellulase from *Trichoderma reesei* (*Celluclast® 1.5 L*, Catalog No. C2730; Sigma-Aldrich, St. Louis, MO, USA) and Laccase from *Trametes versicolor* (Catalog No. 38429; Sigma-Aldrich, St. Louis, MO, USA) at a concentration of 1 unit per gram of grass suspended in sodium acetate buffer at a pH of 4.8. Considering that each gram of grass substrate contains approximately 0.2–0.3 g lignin, the dosage of 1 unit per gram of substrate corresponds to about 5 units per gram of lignin (Hamalainen et al. 2018). Cellulase was chosen as it improves saccharification of cellulose, which makes it more easily accessible to the microbes and thereby reduces the recalcitrance of cellulose. 10 g of grass was added to 100 ml of the enzyme cocktail, stirred at 150 rpm, and incubated at 55 °C for different durations. Each batch was removed after 12, 24, 36, and 48 h, respectively.

### Impact of stirring

To evaluate the influence of stirring on treatment efficacy, the optimal concentrations and duration identified for chemical and ultrasonic treatments were selected for further testing. Enzymatic pretreatments inherently involve shaking or incubation to maintain homogenous enzyme-substrate interactions and facilitate diffusion, making separate evaluation of stirring redundant. By restricting the evaluation to the treatments with the highest preliminary lignin removal, the study optimizes resource utilization, reduces energy and time expenditure, and ensures that the mechanistic contribution of stirring is quantified only for the most promising treatment protocols. Based on superior performance in the initial trials, 0.9% NaOH, 1% H_2_SO_4_, and 22% Ammonia treatments were chosen. This approach allows for a clear delineation of the role of mechanical agitation in enhancing lignin disruption, independent of the inherent chemical or ultrasonic effects. Additionally, the effect of stirring on the distribution of cavitation efficiency during ultrasonication can also be examined. Each experiment was conducted in duplicate, with and without mechanical stirring at 250 rpm using a magnetic stirrer.

For each trial, 10 g of substrate were immersed in 100 ml of the chemical solution. The preparation of chemical solutions adhered strictly to the procedures detailed in Section “Treatment of grass”. Following a 24-h treatment period, the substrates were systematically filtered using a sieve to ensure consistent sample recovery. This experiment was designed to isolate the effect of stirring and evaluate its specific contribution to lignin reduction during the treatment process. Additionally, Welch’s t-test was performed to compare the lignin concentrations of unstirred and stirred samples in order to evaluate the statistical significance of stirring on lignin reduction.

### Analytical methods

#### Fractionation of lignin, cellulose, and hemicellulose

The fractionation was performed by following the Van Soest fiber analysis protocol (Van Soest et al. 1991). The ground and dried samples were refluxed for 1 h in the neutral detergent solution containing sodium lauryl sulphate, disodium ethylenediamine tetraacetate, sodium borate decahydrate, 2-ethoxy ethanol, and disodium hydrogen phosphate, followed by washing with acetone, filtration, and drying at 100 °C for 8 h. The dried content is called neutral detergent fiber, which was weighed. In the next step, the air-dried sample was refluxed for 1 h in the acid detergent solution containing trimethyl ammonium bromide mixed in a diluted sulfuric acid solution. The refluxed sample was washed with acetone till no further color was removed and filtered, followed by vacuum filtration and drying at 100 °C for 24 h. The value of acid detergent fiber was calculated from this step by including a correction factor of 0.9. The final step involved the suspension of acid detergent fiber in 72% sulphuric acid for 3 h, followed by washing and filtration to measure the acid detergent lignin. The proportions of lignin, cellulose, and hemicellulose were determined using Eqs. (1), (2), and (3).

1 $$ {\text{Hemicellulose}} = {\text{Neutral Detergent Fiber}} - {\text{Acid Detergent Fiber}} $$

2 $$ {\text{Cellulose}} = {\text{ADF}} - {\text{Residue after extraction with }}72\% {\text{H}}_{2} {\text{SO}}_{4} $$

3 $${\text{Lignin}}={\text{Residue after extraction with 72}}\% {{\text{H}}_{\text{2}}}{\text{S}}{{\text{O}}_{\text{4}}}-{\text{Ash}}$$

#### Structural and morphological characterization

The structural and morphological characterization of the samples with maximum lignin reduction in each treatment set was undertaken due to the large number of samples.

##### Scanning electron microscopy

SEM Analysis was performed to understand the morphological effects of treatment. A representative sample was chosen for the analysis, and the fibers were separated and mounted on a carbon tape stuck on a conductive stub. The sample was sputtered to avoid charging during the scanning. A Field Emission Scanning Electron Microscope (FESEM) of FEI make (Model: Apreo LoVac) was used. The magnification scale was kept at 10,000, at an accelerating voltage of 20 kV and a working distance of 10 mm.

##### Fourier transform infrared spectroscopy

The effect of treatment on various types of bonds between lignin, cellulose, and hemicellulose was analyzed using the Bruker FTIR Alpha Spectrometer over a scanning range of 500–4000 cm^−1^ by directly placing the sample on the diamond crystal plate. The analysis was done using Opus software.

##### X-ray diffractometry

The crystallinity assessment was conducted using a Rigaku Ultima IV X-ray diffractometer (XRD). The X-ray tube was operated at 40 kV and 30 mA, and the K-beta filter was used to monochromatize the X-ray beam. The scanning speed was 5° per minute, and the 2θ scanning range was from 5° to 80°.

### Severity factor

The formulation of the combined severity factor (CSF), as established by Pedersen and Meyer (2010), is mathematically represented in Eqs. (4) and (5):

4 $$ {\text{CSF}} = \log {\text{ R}}_{o} + |{\text{pH}} - 7| $$

5 $$ {\text{R}}_{{\text{o}}} = t \times \exp ~\left( {\frac{{T - 100}}{{14.75}}} \right) $$

where t denotes the reaction time (min), T represents the reaction temperature (°C), and pH corresponds to the pH of the treatment slurry. This combined severity, defined by Pedersen and Meyer (2010), incorporated the effect of pH along with the temperature and duration associated with the conventional severity factor as defined by Overend, Chornet, and Gascoigne (1987).

### Specific energy

The specific energy input (E_s_) was determined based on ultrasonic power, substrate volume, and solids concentration, as described by Eq. (6) (Marta et al. 2020).:

6 $$ {\text{E}}_{{\text{s}}} = \frac{{P \times t}}{{V \times TS^{\prime}}} $$

where P denotes the applied ultrasonic power (kW), t is the duration of sonication (s), V represents the volume of the substrate (L), and TS′ is the volatile solids concentration (kg/L).

### Cost analysis

The cost analysis was conducted to provide a comprehensive understanding of the economic factors involved in various treatments, focusing on their cost-effectiveness in reducing lignin content. The total cost calculation encompassed several components: the cost of chemicals, distilled water, energy consumption, and daily equipment depreciation (Guan et al. 2025). Total Treatment Cost (USD/kg of substrate) can be defined as the sum of expenditures on chemicals, distilled water, energy, and equipment depreciation as given in Eq. (10). The energy cost was calculated using Eq. (7) Cost per Percentage Reduction in Lignin was assessed by dividing the total treatment cost by the percentage reduction in lignin achieved, providing a measure of cost-effectiveness specific to lignin removal as given in Eq. (8). Equipment Depreciation (USD/day) was calculated based on the purchase price of the equipment, estimated lifespan, and daily operation, offering insights into the long-term economic impact of equipment wear and tear as given in Eq. (9).

7 $$ \begin{gathered} {\text{Cost of energy}} = {\text{power rating of the equipment }}\left( {{\text{in kW}}} \right) \hfill \\ \;\;\; \times {\text{ duration of usage of equipment }}\left( {{\text{in hours}}} \right) \hfill \\ \;\;\; \times {\text{ energy cost in the region }}\left( {{\text{in USD per kWH}}} \right) \hfill \\ \end{gathered} $$

8 $$ \begin{gathered} {\text{Cost per percentage reduction in lignin}} \hfill \\ \;\;\;\;\; = \frac{{{\text{cost~of~treatment~per~kg}}}}{{\% ~of~lignin~reduction}} \hfill \\ \end{gathered} $$

9 $$ \begin{gathered} {\text{Cost of equipment depreciation }}\left( {{\text{per day}}} \right) \hfill \\ \;\;\;\;\; = \frac{{cost~of~equipment}}{{useful~life~in~days}} \hfill \\ \end{gathered} $$

10 $$ \begin{gathered} {\text{Cost of treatment }}\left( {{\text{per kg of substrate}}} \right){\text{ USD}} \hfill \\ \;\; = {\text{cost of chemical}} + {\text{cost of water}} + {\text{cost of energy}} \hfill \\ \;\;\;\; + {\text{cost of equipment depreciation per day}} \hfill \\ \end{gathered} $$

The useful life of all the equipment was assumed to be 5 years. The energy cost was assigned based on the energy tariff of the Government of Telangana, India i.e., Rs.11/kWH, (USD 0.132/kWH). The cost of distilled water was assigned based on the previous literature as Rs.1.5/liter (USD 0.018/liter) (Reddy and Sharon 2018). The cost of chemicals was assigned based on the pricing given on the product website. By evaluating energy consumption, chemical use, water usage, and equipment depreciation, this analysis offers a holistic view of the economic implications. Moreover, calculating the cost per percentage reduction in lignin provides a targeted metric for assessing the cost-effectiveness of different treatments, ensuring that economic evaluations align closely with performance outcomes.

### Statistical significance

The Welch’s t-test was used to determine the statistical significance of the treatment. Unlike the standard Student’s t-test, Welch’s t-test does not assume equal variances or equal sample sizes between groups, making it more robust for experimental data where pretreatment effects may alter variability in unpredictable ways. In this study, the assumption of unequal variance is valid because different pretreatments can cause highly variable reductions in lignin concentration depending on the substrate-treatment interactions. A one-tailed *p*-value is used for the analysis at a 95% confidence interval. This is because the lignin concentration is expected to decrease after treatment. It is not feasible for the treatment to increase the lignin content beyond that of the untreated sample. The lignin values of each treatment set were compared to that of the untreated sample’s lignin value, and the *p*-values were calculated.

## Results and discussion

### Impact of treatment on lignin, cellulose, and hemicellulose composition

**Fig. 3:**
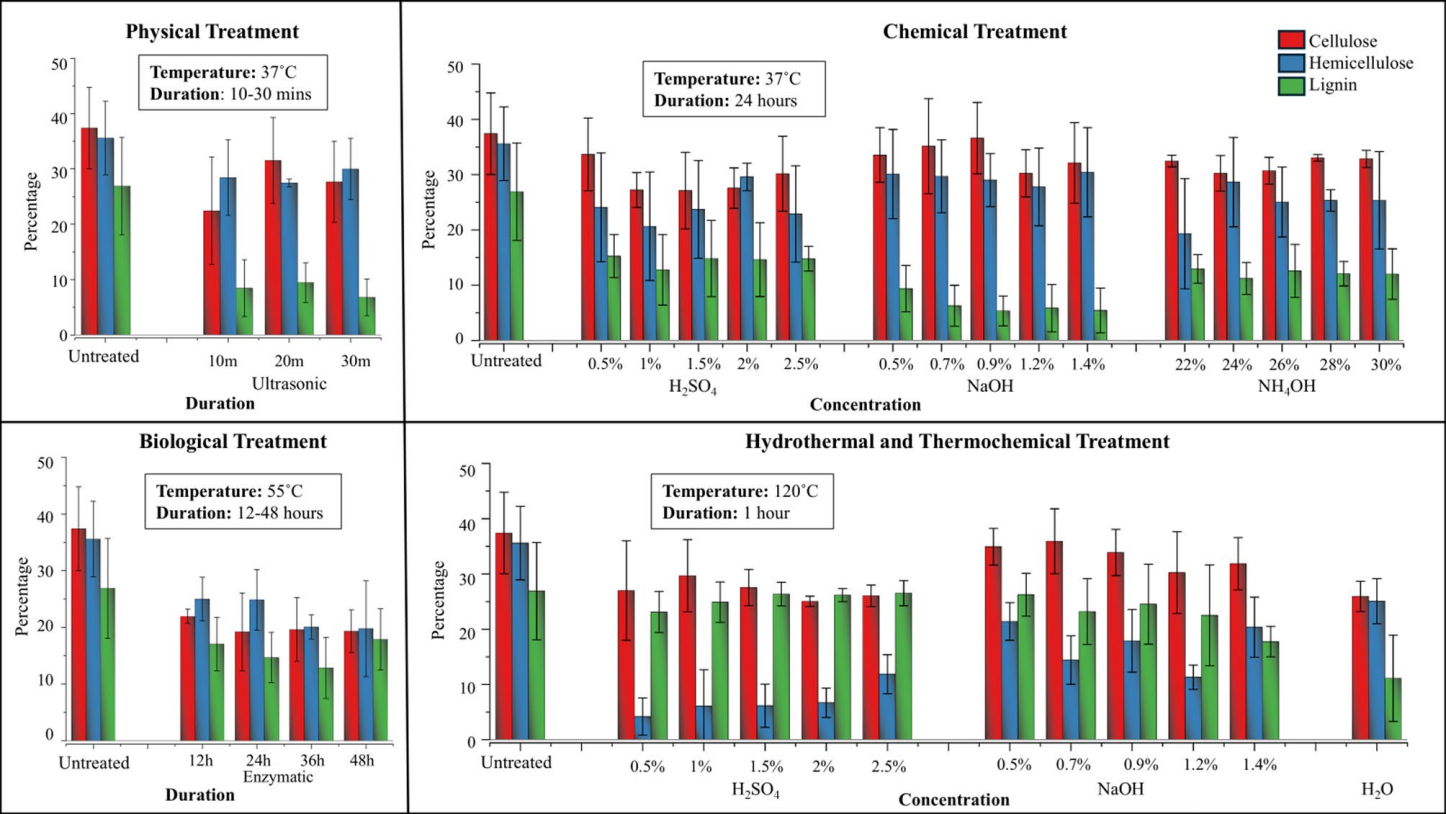
Lignin, cellulose, and hemicellulose compositions of untreated and pretreated grass clippings

The fractionation of untreated grass clippings showed that it consisted of 32% lignin, 39% cellulose, and 29% hemicellulose, excluding the extractives content. Since the treatment affects the percentage of extractives, to provide a more accurate comparison of the impact of various treatments on the structural components, the extractives content was excluded. This approach allowed for a normalized analysis of the lignin, cellulose, and hemicellulose fractions, thereby offering a clearer representation of the treatment impact on the key constituents of grass clippings. The fractionation results of the grass clippings post-treatment are plotted below in Fig. 3.

Fractionation of all 34 samples was done in triplicate, and the measured average and standard deviation values were plotted. The main aim of treatment was to remove lignin and reduce the recalcitrance of grass clippings while retaining the cellulose and hemicellulose for efficient valorization. Almost all the applied treatment methods led to a reduction in lignin content, while only a few were able to retain the cellulose and hemicellulose contents.

Alkaline solutions, including sodium hydroxide and aqueous ammonia, disrupt cellulose crystallinity and cleave acetyl linkages within the cell walls, facilitating lignin removal. *The alkaline pretreatments at room temperature also* showed low variance across replicates, indicating high reproducibility and robustnes*s*. However, during combined alkaline and heat treatment, biomass condensation restricts sodium hydroxide penetration, preventing significant lignin reduction in HPHT treatment.

Sulphuric acid can also cleave β-O-4 ether bonds and disrupt cell structure, leading to methoxy bond cleavage and increased hydroxyl group concentration, corroborated by FTIR results. Sulphuric acid treatment at HPHT led to a loss of nearly 30–40% of the biomass by weight compared to the untreated grass, of which a major portion was hemicellulose. Notably, sulphuric acid treatment at HPHT exhibited the highest variance among replicates, indicating lower reproducibility and suggesting that small fluctuations in reaction conditions strongly influence the treatment outcome. Heat is known to solubilize hemicellulose, explaining the increased hemicellulose loss in the treatments conducted at HPHT.

During ultrasonication, the high-frequency sound waves lead to alternating high-pressure and low-pressure zones, which disrupt the cells through cavitation. This has led to a significant reduction in the lignin content. Degradation of lignin by the action of the enzyme laccase has led to the reduction of lignin. While cellulase was included to reduce the recalcitrance of cellulose, it also degraded a considerable portion of the cellulose. Laccase targets phenolic structures in lignin, while cellulase non-selectively hydrolyses amorphous regions, which is why cellulose losses were higher. Overall, 0.9% sodium hydroxide treatment at 37 °C demonstrated superior lignin removal while effectively preserving cellulose and hemicellulose integrity, followed by ultrasonic treatment.

### Percentage reduction of lignin

**Fig. 4:**
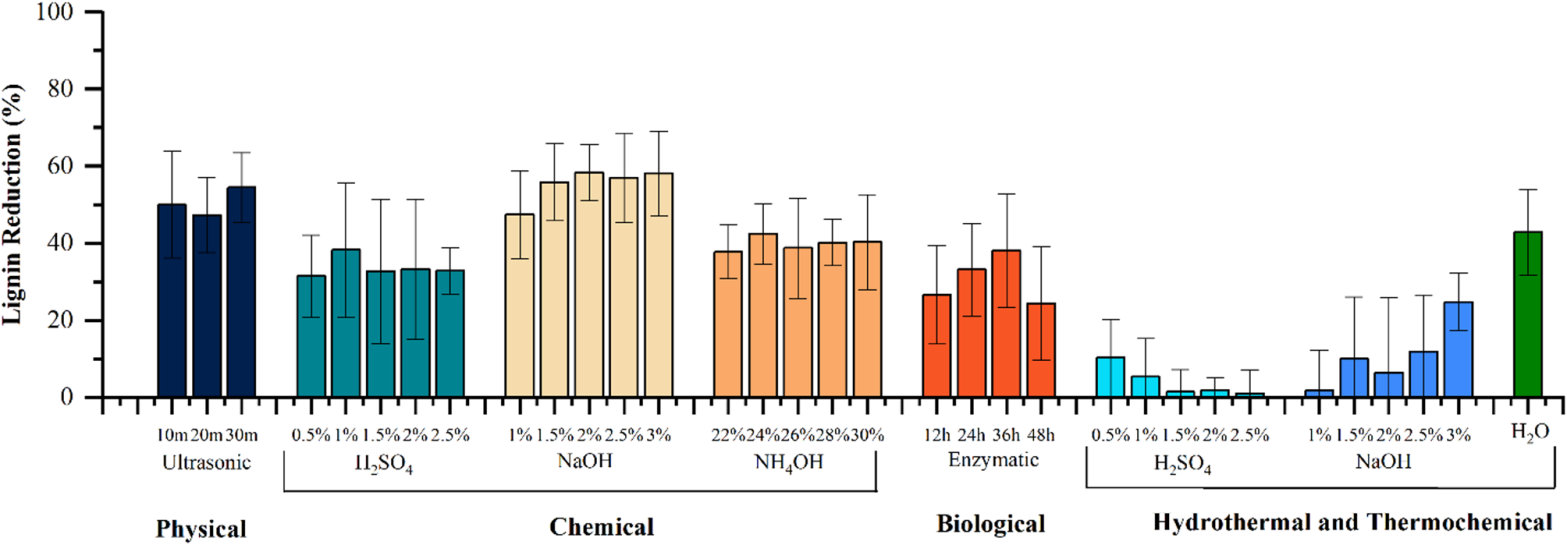
Lignin reduction caused by various treatments, when compared to untreated grass clippings

As lignin is considered the key factor causing the recalcitrance in lignocellulosic compounds, specific focus has been given to analyzing the percentage of lignin reduction caused by various treatments. It can be observed from Fig. 4 that the highest percentage of lignin reduction, 58.37%, occurred during sodium hydroxide treatment at 37 °C. The next highest reduction was caused by ultrasonic treatment at 54.52%. Heat treatment led to a 42.8% reduction; however, sulphuric acid and sodium hydroxide treatment at HPHT led to a very minimal reduction. The sulphuric acid treatment and aqueous ammonia treatment at 37 °C led to a 30–40% reduction in lignin, followed by the enzymatic treatment.

### Analysis of treatment slurry

**Fig. 5:**
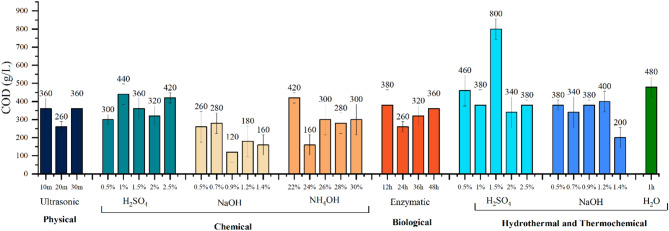
COD concentrations of the treatment slurry

The Chemical Oxygen Demand (COD) results of the treatment slurry were consistent with the overall biomass loss percentage observed for the lignin, cellulose, and hemicellulose fractions. The sulphuric acid treatment at HPHT resulted in the highest biomass loss, which correlates with the high COD concentration in the slurry, shown in Fig. 5.

The COD values of all other treatment slurries ranged from 200 to 400 g/L, confirming that the lost biomass had been solubilized. To ensure reproducibility, all COD measurements were performed in triplicate, and the standard deviation across replicates was found to be within 6–22% of the mean, indicating good consistency of results.

### Impact of stirring on lignin reduction

**Fig. 6:**
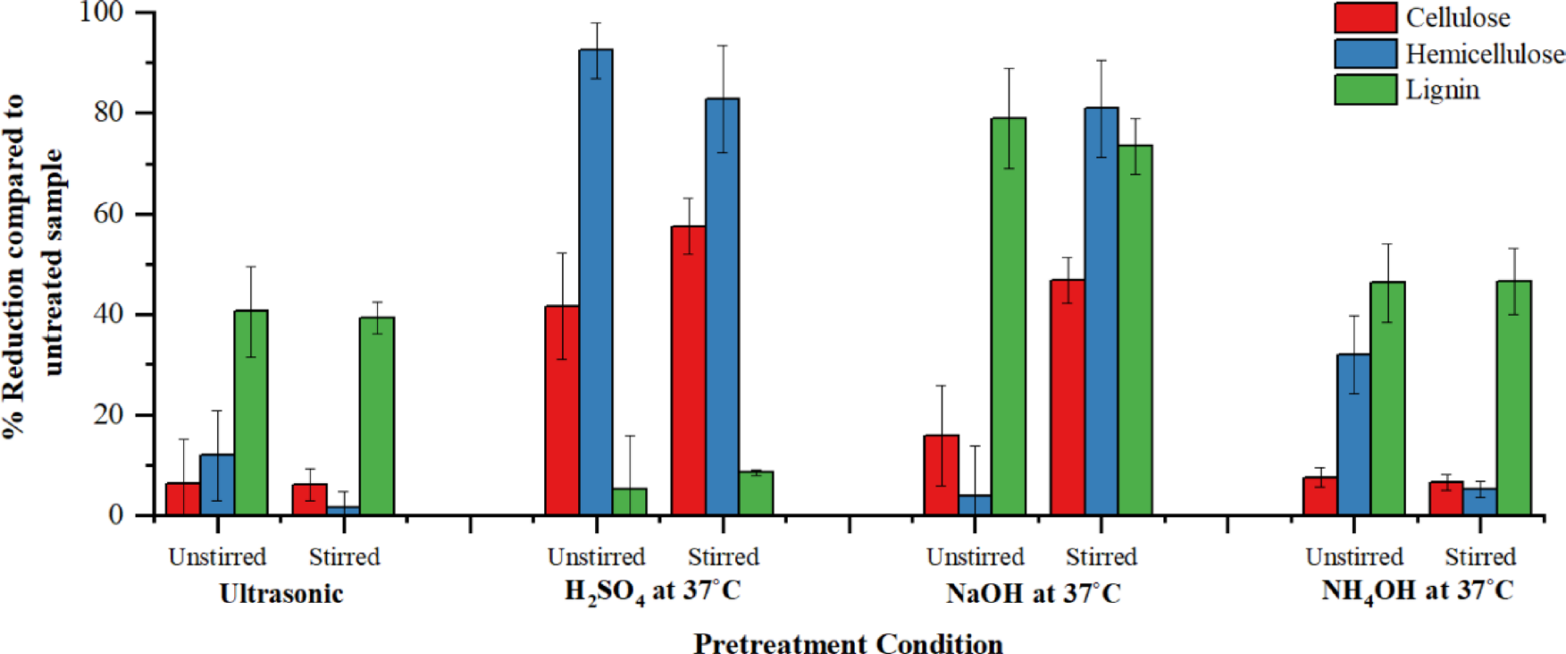
Reduction of lignin, cellulose, and hemicellulose when compared to untreated grass clippings for stirred and unstirred treatment conditions

The results of lignin, cellulose, and hemicellulose estimation pre- and post-treatment with and without stirring showed that the percentage reduction of lignin was not significantly impacted by stirring. The percentage reduction of lignin in ultrasonic treatment with stirring showed that the sample without stirring had a 40.58% reduction in lignin compared to the untreated sample, whereas the one with stirring had only a 39.32% reduction. While the difference is small, it can be clearly seen from Fig. 6 that stirring did not have any impact on the effectiveness of ultrasonic treatment. Similarly, Welch’s t-test (test statistic = 0.041, df = 6, *p* = 0.969) confirmed that the difference was not statistically significant, and the two groups were statistically indistinguishable. The close variances of 0.0048 (stirred) and 0.0061 (unstirred) further support that mechanical stirring did not introduce variability or measurable effects under the tested conditions.

The results of alkaline treatment showed that not only was the lignin reduction percentage lower in the sample with stirring, but it also led to a significant loss of cellulose and hemicellulose. This combination can be utilized if the main aim is to completely solubilize the biomass. The acid treatment showed a 3.27% increase in lignin reduction with stirring, whereas the loss of cellulose increased, and the loss of hemicellulose decreased. The ammonia treatment showed that the lignin reduction percentage was almost the same with and without stirring, with the reduction of cellulose and hemicellulose decreased by 1% and 25%, respectively.

### Structural, morphological, and compositional analysis

#### Scanning electron microscopy analysis

**Fig. 7:**
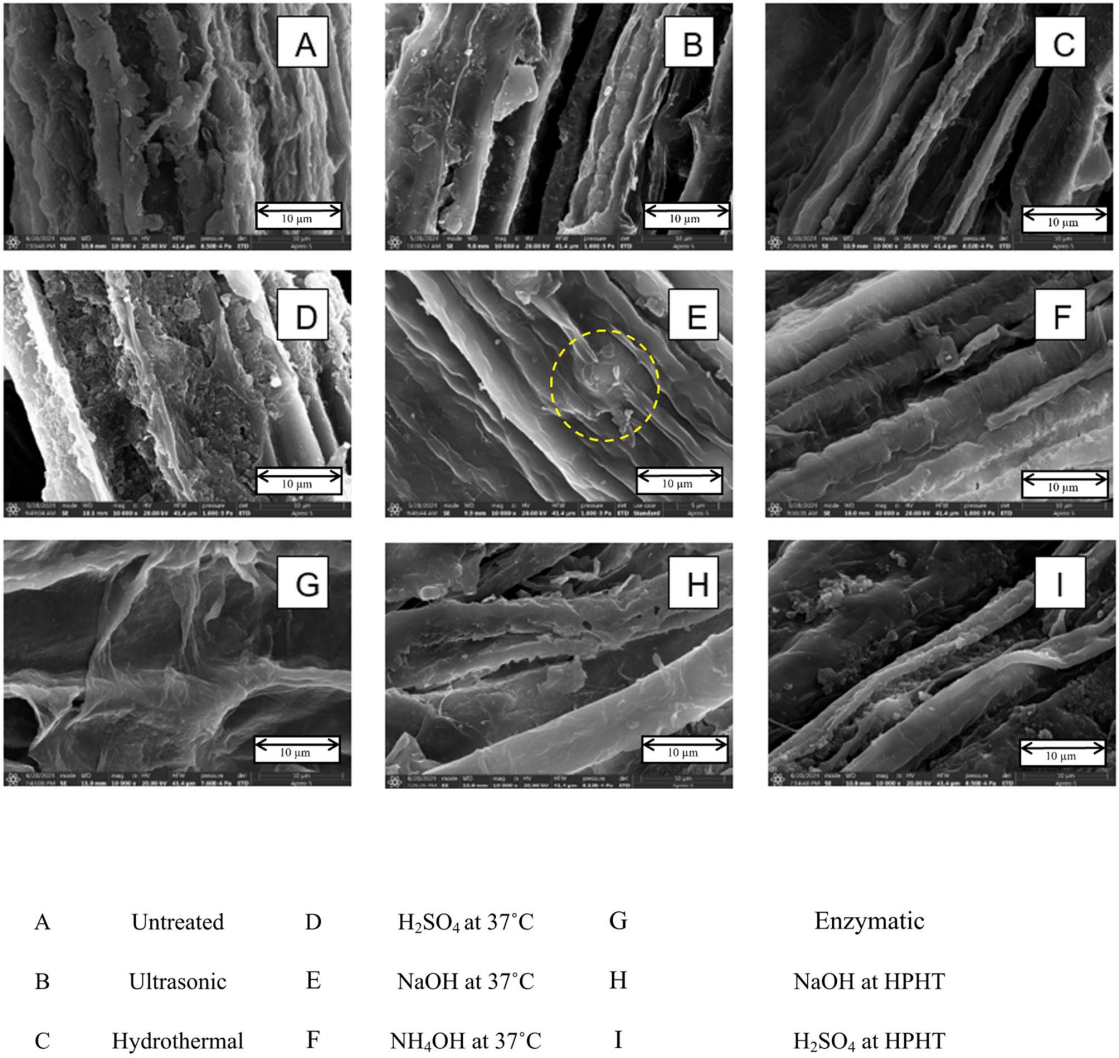
SEM images of untreated and treated grass clippings

Figure 7 shows the SEM images of untreated and pretreated grass clippings. The surface of the untreated sample (Fig. 7A) appears rough and fibrous, with various irregular structures. The differences in morphology across the treatments demonstrate that each treatment had a unique effect, as detailed below. Ultrasonic (Fig. 7B) and hydrothermal (Fig. 7C) treatments reduced the appearance of loose debris, and the fibers appeared more defined. Sulphuric acid treatment at 37 °C (Fig. 7D) caused increased disruption of the cell structure, resulting in the formation of more pores and cavities. Similarly, hydrothermal treatment (Fig. 7C) also led to a cleaner appearance, with reduced loose debris.

Sodium hydroxide treatment at 37 °C caused saponification, which led to swelling and disruption of the cellular structure, resulting in a smoother surface, as circled in Fig. 7E. Aqueous ammonia treatment (Fig. 7F) led to noticeable shrinkage of the fiber surfaces. Sodium hydroxide treatment at high pressure and high temperature (HPHT) (Fig. 7H) produced morphological changes similar to those observed in the hydrothermal treatment, but with increased fiber disruption.

Enzymatic treatment (Fig. 7G) resulted in increased surface roughness while also reducing the appearance of loose debris. Finally, sulphuric acid treatment at HPHT (Fig. 7I) showed the most aggressive breakdown of structure, characterized by significant pore formation and fragmentation. Among all treatments, sodium hydroxide at 37 °C (Fig. 7E) showed the most effective disruption and smoothening of the cell structure, indicating its superior performance for enhancing surface accessibility.

#### FTIR spectroscopy analysis

**Fig. 8:**
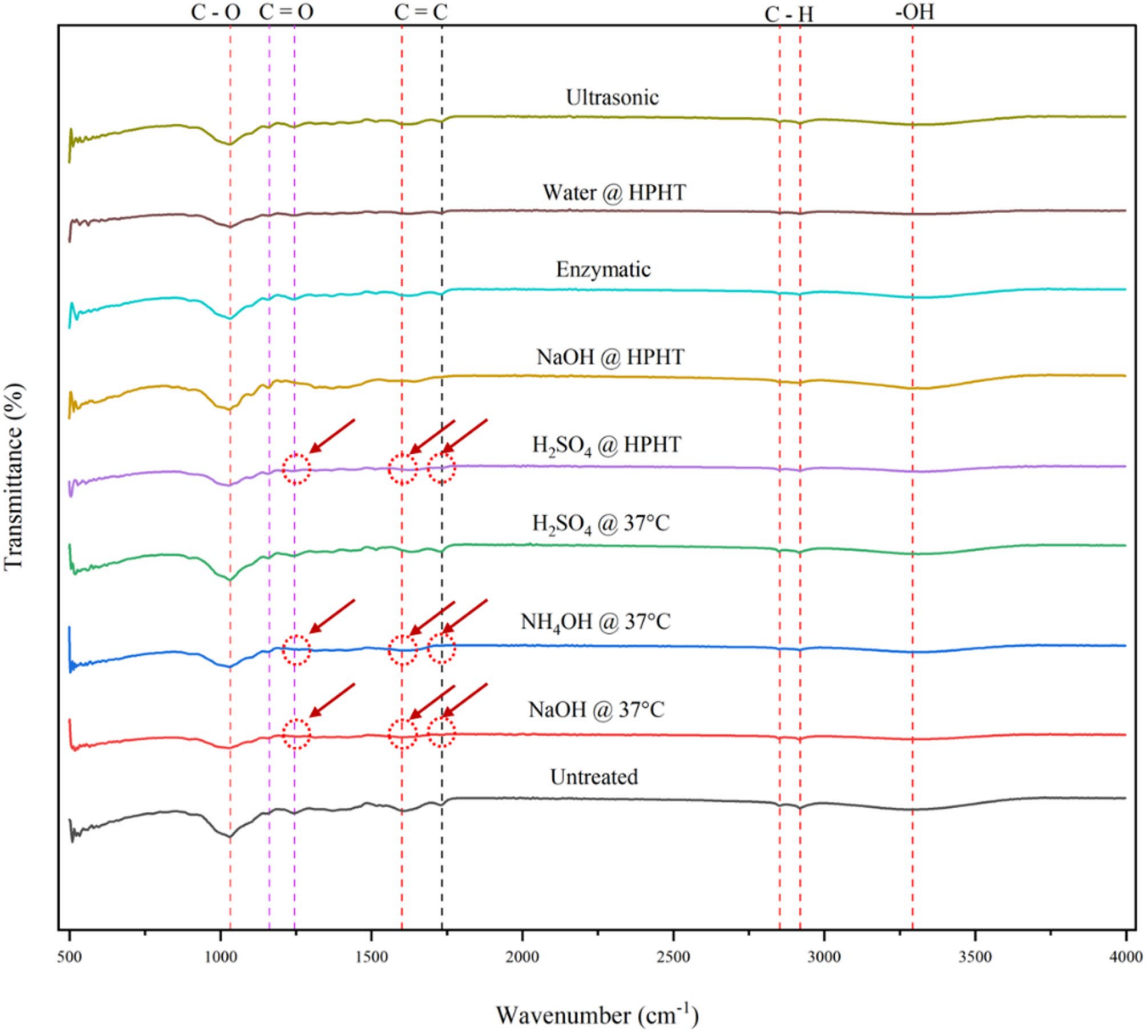
FTIR spectroscopy images of untreated and pretreated grass clippings

The peaks found in the untreated grass are given below in Table 1. It can be observed that there are no significant changes in the peaks in the sodium hydroxide treatment at HPHT, sulphuric acid treatment at 37 °C, and ultrasonic and enzymatic treatments when compared to the untreated sample. In the hydrothermal treatment, the peak at 1605 cm^− 1^ completely disappeared, but the peak at 1735 cm^− 1^ persisted, indicating that the lignin is bonded with hemicellulose. In the sodium hydroxide treatment at 37 °C, aqueous ammonia treatment at 37 °C, and sulphuric acid treatment at HPHT, the peaks at 1605 cm^− 1^ and 1735 cm^− 1^ disappeared, breaking the lignin-based bonding, as highlighted in Fig. 8.

**Table 1:** Important wavelengths corresponding to the characterization of lignin, cellulose, and hemicellulose

Wavenumber (cm^− 1^)	Bond	Significance
1033, 1162, 1245	C–O	Linked to cellulose and hemicellulose
1605	C=C	Associated with lignin
1735	C=O	Associated with ester linkages in lignin or hemicellulose.
2854, 2924	C–H	Observed in plant lipid
3294	O–H	Cellulose, hemicellulose, or other hydroxyl-containing compounds.

The disappearance of the peak at 1605 cm^− 1^ (C=C in lignin) in sodium hydroxide at 37 °C, aqueous ammonia at 37 °C, and sulphuric acid at HPHT indicates cleavage of aromatic lignin structures, confirming delignification. Retention of the 1735 cm^− 1^ peak in hydrothermal treatment suggests that ester linkages between lignin and hemicellulose remain partially intact, explaining why lignin removal was less pronounced in this case. The minimal change in peak intensity for ultrasonic and enzymatic treatments reflects selective action, whereas mechanical disruption or enzymatic hydrolysis targets surface lignin without extensive backbone cleavage.

#### X-ray diffraction (XRD) analysis for crystallinity

Due to the amorphous nature of the grass samples, the XRD analysis formed flat and wide peaks. It can be observed that there was no significant structural change in crystallinity during the ultrasonic treatment.

**Fig. 9:**
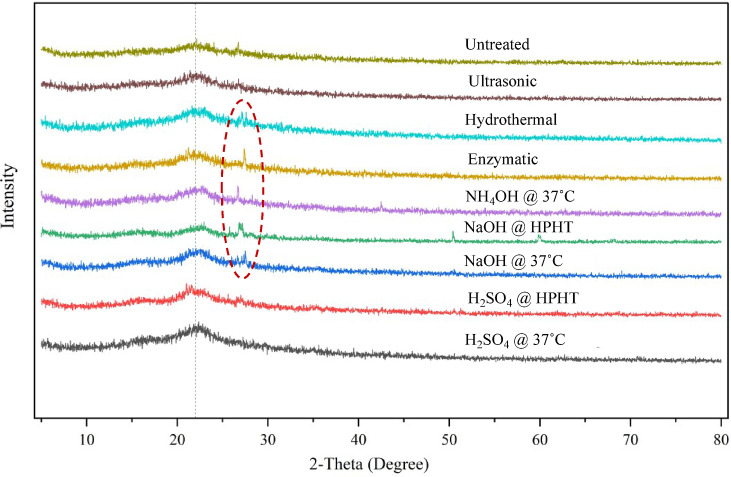
XRD Diffractograms of untreated and pretreated samples

The peak was observed at 22.88° for the untreated sample, whereas there was a slight shift to 22.20° during ultrasonic and hydrothermal treatments, which is negligible. New peaks were found at around 26–27° in enzymatic, aqueous ammonia treatment at room temperature, sodium hydroxide treatment at HPHT, and sodium hydroxide treatment at room temperature, as highlighted in Fig. 9, indicating partial rearrangement of cellulose microfibrils or formation of disordered crystalline domains due to extensive lignin removal and fiber swelling.

Sodium hydroxide treatment at HPHT showed additional peaks at 50.397 and 59.868°. The appearance of new peaks denotes an increase in the degree of structural disorder, which is consistent with the findings of the FTIR analysis. Such peak appearances are consistent with FTIR evidence of aromatic C=C bond cleavage and confirm disruption of lignin-cellulose matrices. The peak has shifted to 21.4° during sulphuric acid treatment at room temperature and at HPHT, indicating that the interplanar spacing has increased, likely due to hydrolytic cleavage of hemicellulose and lignin, corroborating SEM observations of larger pore formation and fragmentation. It is consistent with the SEM images, where there was an appearance of larger pores as compared to the untreated sample. The absence of sharp peaks indicates that the grass clippings remained amorphous after treatment.

### Combined severity factor analysis

**Fig. 10:**
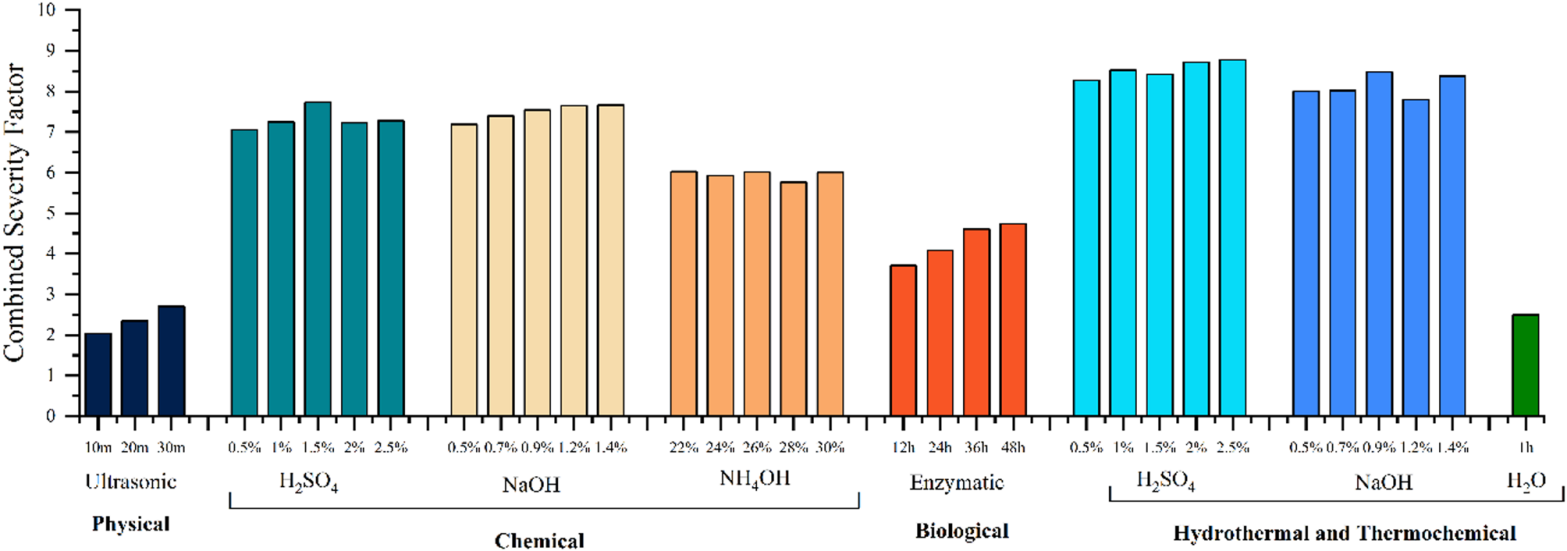
Combined Severity Factor of various treatments of grass clippings

The results of the combined severity factor, as shown in Fig. 10 below, indicate that acid and alkaline treatments had significantly higher severity compared to the hydrothermal, ultrasonic, and enzymatic treatments. However, as the combined severity factor does not consider the specific energy of the ultrasonic treatment and the enzymatic activity, the severity is solely based on the pH, temperature, and duration. Hence, the results are consistent for chemical, hydrothermal, and thermochemical treatments, but do not comprehensively reflect the results of ultrasonic and enzymatic treatments.

### Cost analysis

As described earlier, the cost calculation was done by combining the cost of chemicals, distilled water, energy consumption, and daily equipment depreciation. The results are presented in Tables 2, 3 and 4, and 5 as heat maps, where red indicates high cost, yellow indicates moderate cost, and green represents the lowest cost. Based on the results, stirring increases the treatment cost by more than 4 times in acid and alkaline treatment, but the lignin reduction was better without stirring. Overall, it can be observed that the hydrothermal treatment without stirring and the 0.5% NaOH treatment at 37 °C without stirring are the most economical treatments. The enzymatic treatment is most expensive due to the cost of commercial enzymes and low lignin reduction. Though the hydrothermal treatment is less expensive, it involves the purchase of equipment and concerned maintenance, and the lignin reduction is not as high as alkaline treatment. To conclude, the 0.5% NaOH treatment at 37 °C without stirring has the highest level of lignin reduction at the lowest cost. The calculated costs are presented on a per-kilogram-of-substrate basis, providing a generalized metric that can be directly extrapolated for larger-scale operations; however, practical implementation at an industrial scale would also require consideration of equipment capacity, chemical recycling, and effluent treatment to ensure feasibility and sustainability.

**Table 2:**
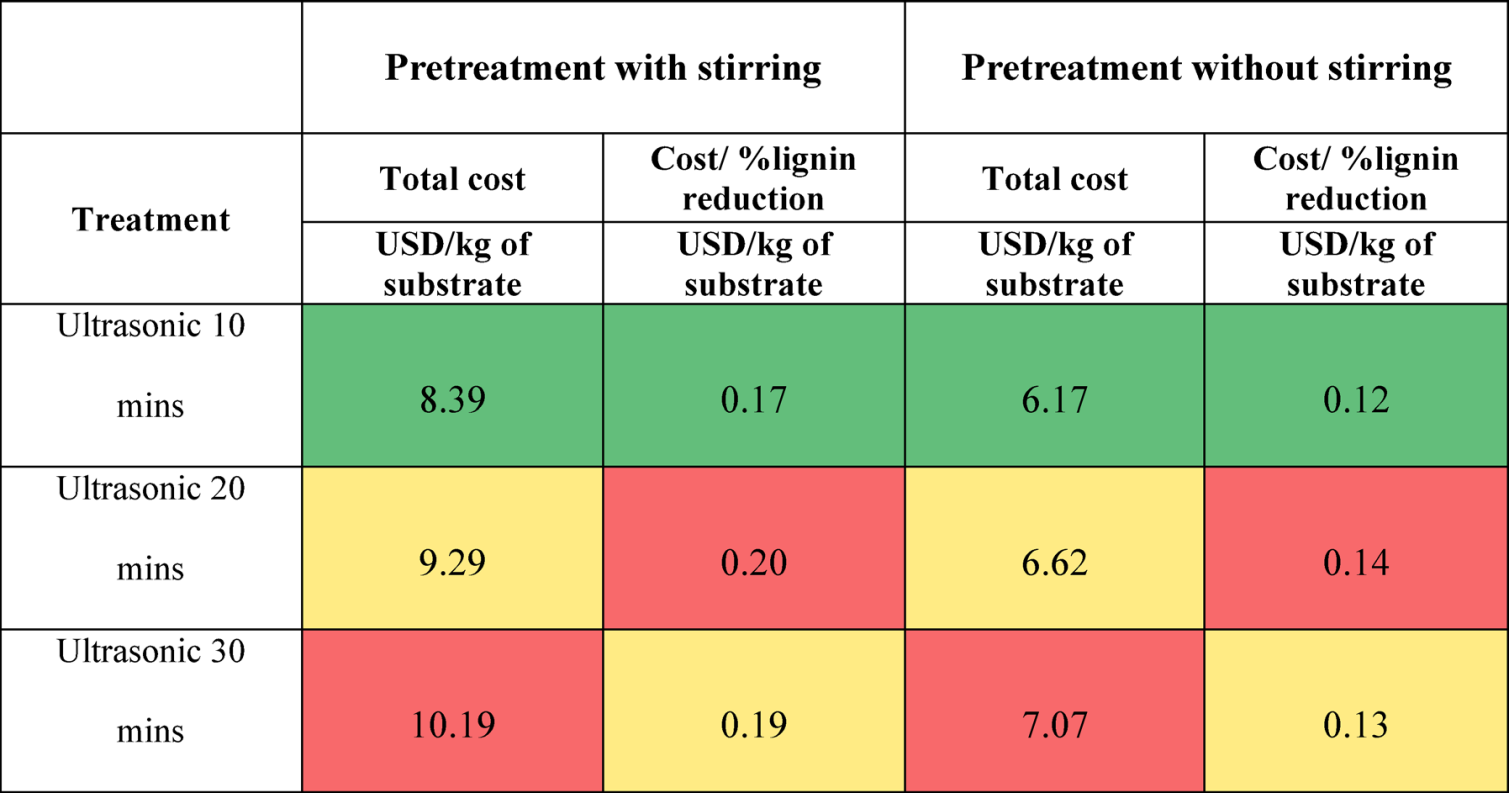
Cost of physical treatment of grass clippings

**Table 3:**
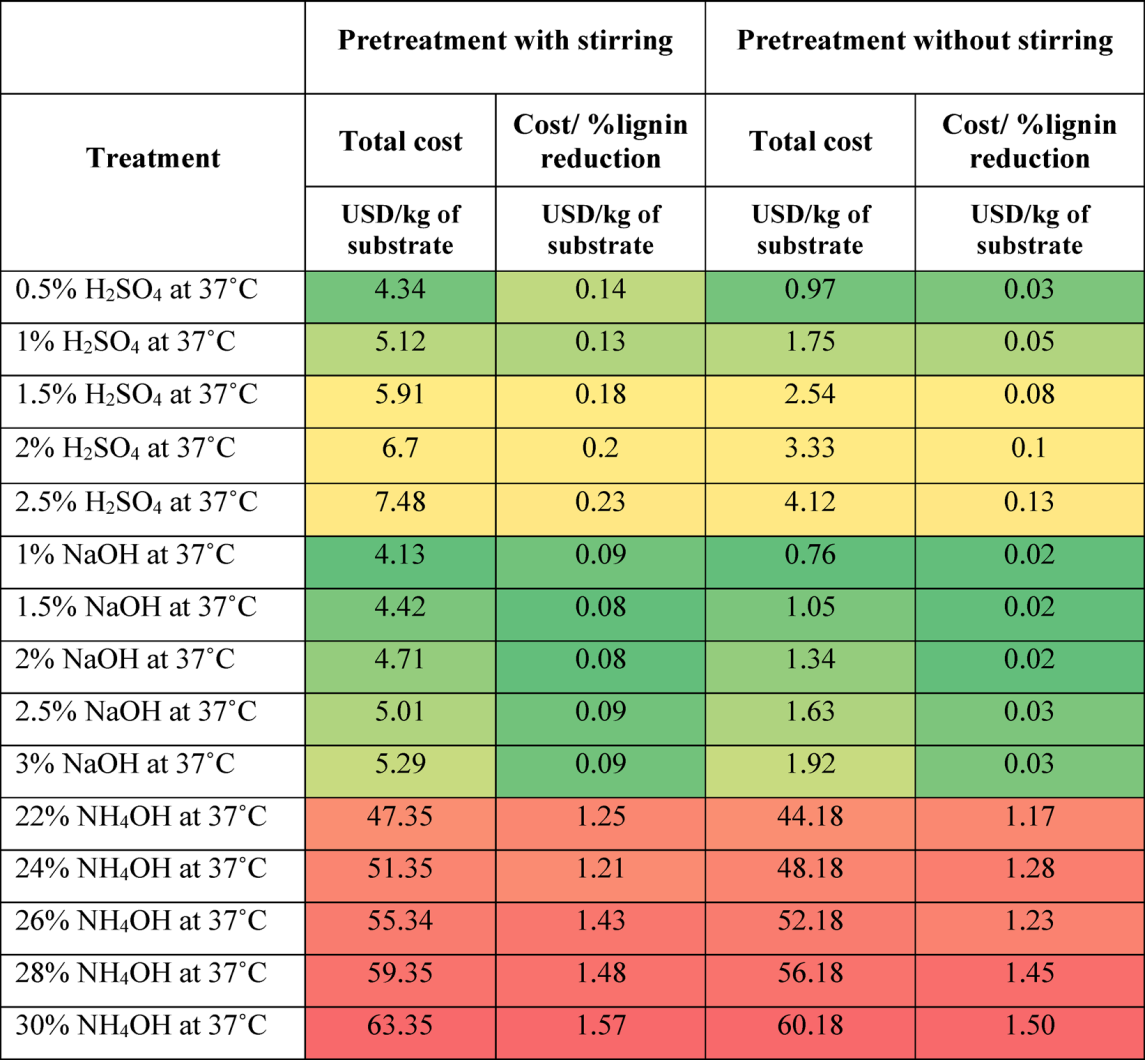
Cost of chemical treatment of grass clippings

**Table 4:**
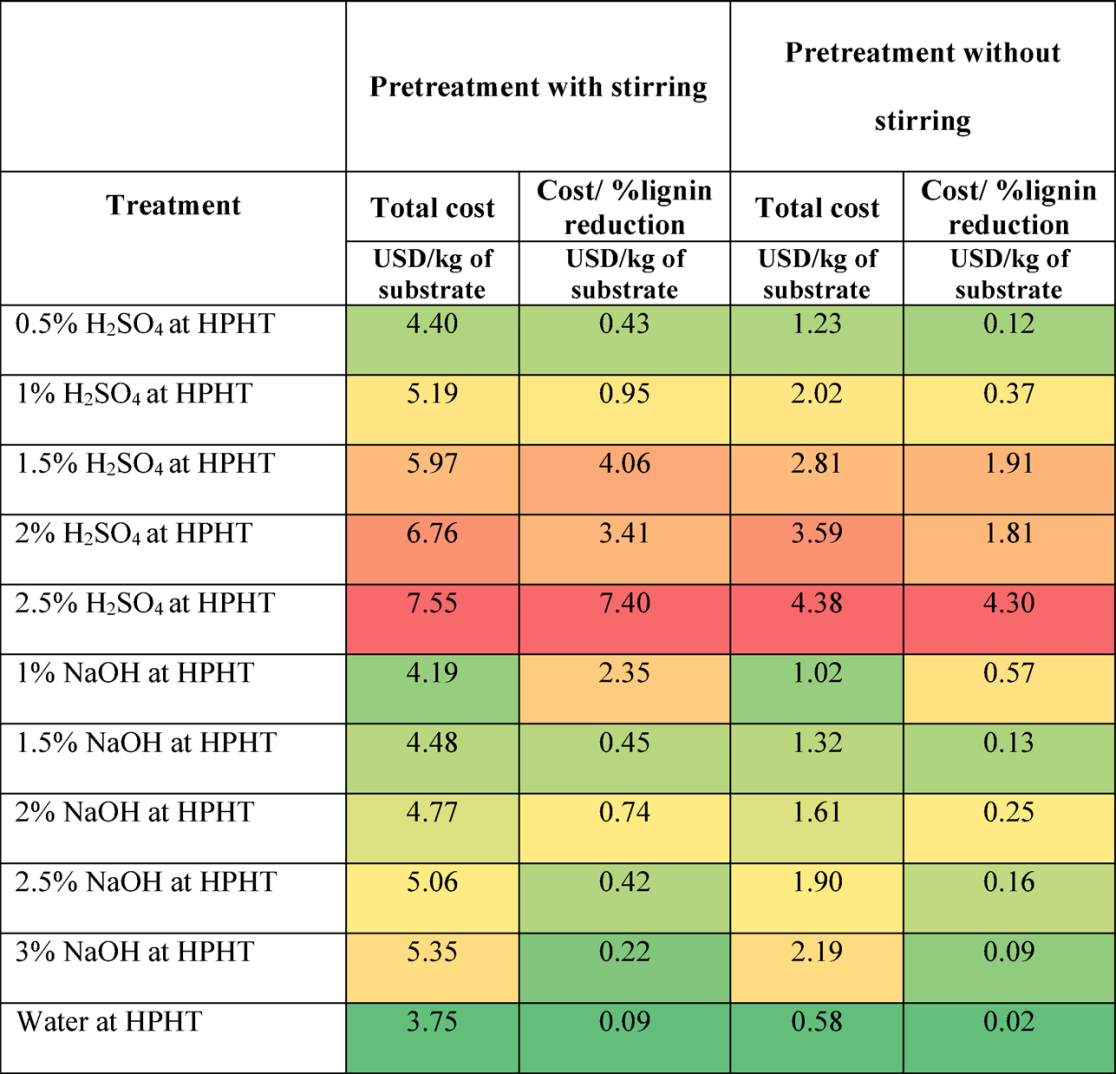
Cost of hydrothermal and thermochemical treatment of grass clippings

**Table 5:**
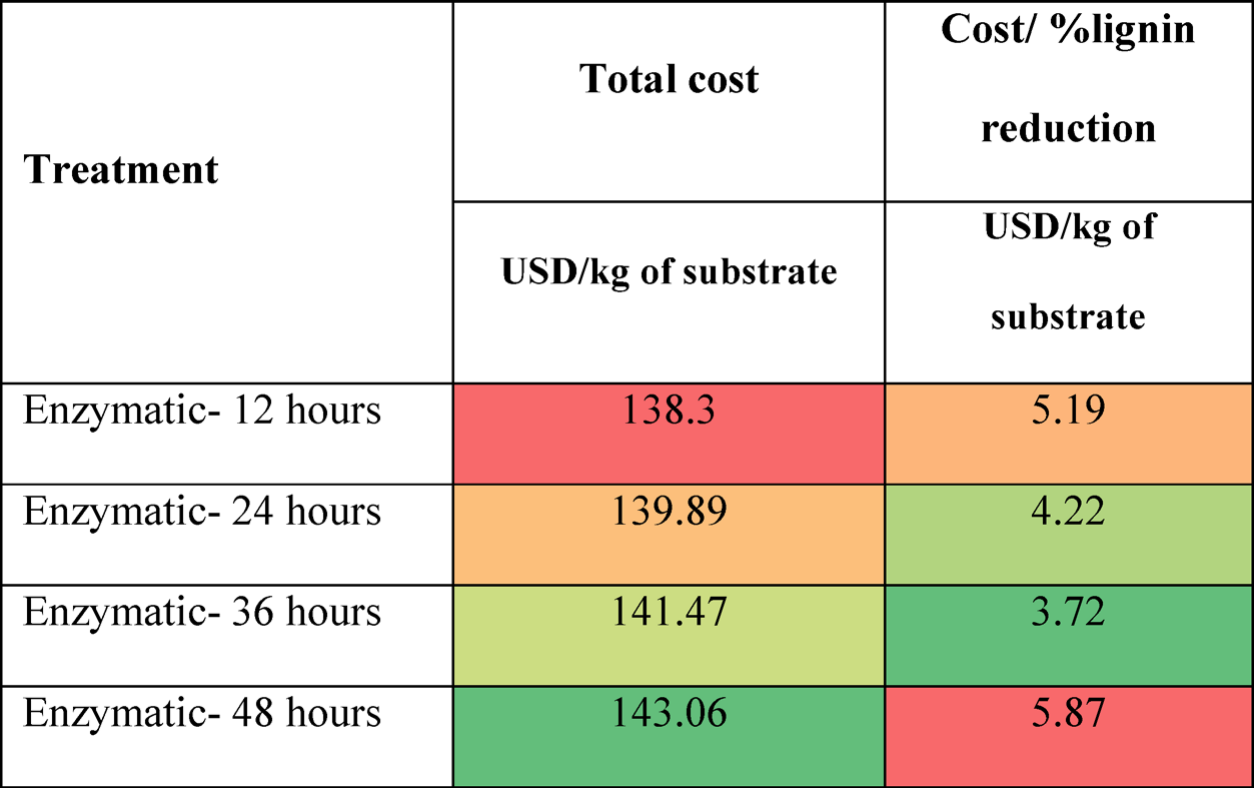
Cost of biological treatment of grass clippings

### Statistical significance

According to the results of the Welch’s t-test given in Table 6, it can be observed that all the *p*-values are less than 0.05 (95% confidence interval), indicating that all the treatments have caused a significant effect on the lignin content. Delving into the order of significance and size of the T-stat, it can be observed that the aqueous ammonia, sulphuric acid, and sodium hydroxide treatments at 37 °C have the lowest *p*-value, indicating that they had the most significant treatment impact. However, the mean values show that the lignin content was lowest in sodium hydroxide treatment at 37 °C.

**Table 6:** Statistical significance of the treatment on the lignin concentrations based on welch’s t-test

Treatment	Mean	Variance	*p*-value	T-stat
H_2_SO_4_ at 37 °C	24.4626	0.9325	4.33 × 10^− 6^	28.7998
NaOH at 37 °C	16.4686	2.8052	5.37 × 10^− 6^	27.2772
NH_4_OH at 37 °C	22.1781	0.4294	4.70 × 10^− 7^	50.2363
H_2_SO_4_ at HPHT	35.4069	2.0826	0.0409	2.3133
NaOH at HPHT	32.8399	10.1441	0.0232	2.8504
Ultrasonic	18.2303	1.8212	0.0009	23.9616
Enzymatic	25.6186	5.2746	0.0011	9.8242

**Table 7:** Statistical significance of the treatment on cellulose and hemicellulose concentrations based on welch’s t-test

Treatment	Cellulose				Hemicellulose			
	Mean	Variance	*p*-value	T-stat	Mean	Variance	*p*-value	T-stat
H_2_SO_4_ at 37 °C	29.1501	7.9333	0.0014	6.5574	24.1896	10.8929	0.0061	4.3363
NaOH at 37 °C	33.5384	6.2357	0.0128	3.4669	29.4042	1.1053	0.0326	2.5219
NH_4_OH at 37 °C	31.8662	1.6708	0.0003	9.5904	24.7493	11.3499	0.009	3.8766
H_2_SO_4_ at HPHT	27.0569	3.0459	9.33 × 10^− 5^	13.2647	6.9869	8.2801	2.60 × 10^− 5^	18.3415
NaOH at HPHT	33.3651	5.3353	0.0087	3.9157	17.0726	17.6232	0.001	7.20007
Ultrasonic	27.2219	20.756	0.0303	3.8733	28.6553	1.5858	0.0585	2.6609
Enzymatic	20.0343	1.6723	5.65 × 10^− 5^	26.8731	22.4297	8.3939	0.0055	5.6331

Regarding cellulose and hemicellulose, the treatment with the least significant impact must be chosen. Since the primary objective of the treatment is to selectively remove lignin while retaining the cellulose and hemicellulose fractions, it is expected that stirring should not cause statistically significant changes in these components. From the *p*-values and T-stat values shown in Table 7, it can be further reiterated that sodium hydroxide treatment at 37 °C and ultrasonic treatment have the least significant effect on cellulose and hemicellulose concentrations. On the other hand, from the mean value, it can be observed that the cellulose and hemicellulose retention is slightly higher in sodium hydroxide treatment at 37 °C than in ultrasonic treatment.

## Conclusion

Lignocellulosic waste, such as grass clippings, can be valorized in various applications, including anaerobic digestion and bioethanol production. The choice of pretreatment should be tailored to the target application: anaerobic digestion prioritizes selective lignin removal to enhance microbial access to cellulose and hemicellulose, whereas bioethanol production benefits from complete biomass solubilization. Alkaline treatment with stirring is effective for solubilization, but in anaerobic digestion, excessive solubilization can accelerate volatile fatty acid accumulation, lowering pH and creating unfavorable conditions for methanogens. This study addressed the knowledge gap of comparing multiple pretreatment methods on a single substrate to identify scalable and application-specific strategies. Some of the key findings are stated below:

- 2% NaOH treatment at 37 °C is the most effective method for lignin reduction, achieving a 58% reduction, and can be easily scaled up without the need for new machinery.
- Ultrasonication significantly reduced lignin, but its scalability remains uncertain.
- Stirring did not significantly enhance lignin removal in chemical and ultrasonic pretreatments, and hence is not required during scale-up, thereby offering potential cost savings.
- NaOH treatment without stirring was identified as the most cost-effective method, outperforming more expensive pre-treatment processes, such as enzymatic treatment, investigated in this study.
- SEM and XRD analyses confirmed that pretreatment improved surface accessibility and maintained amorphous characteristics.

The reusability of slurry generated from NaOH treatment in large-scale biomethanation plants must be explored, especially for pH maintenance without additional investment. Further research is required to optimize the immersion ratio and treatment duration for efficient lignin reduction. This process when integrated into waste management systems, can reduce greenhouse gas emissions and generate value-added products, leading to a circular economy.

## Data Availability

All data generated or analysed during this study are included in this article.
